# Clinical evaluation of a real-time artificial intelligence-based polyp detection system: a US multi-center pilot study

**DOI:** 10.1038/s41598-022-10597-y

**Published:** 2022-04-21

**Authors:** Susan Y. Quan, Mike T. Wei, Jun Lee, Raja Mohi-Ud-Din, Radman Mostaghim, Ritu Sachdev, David Siegel, Yishai Friedlander, Shai Friedland

**Affiliations:** 1 Stanford University, Stanford, CA USA; 2 Veterans Affairs Palo Alto Health Care System, Palo Alto, CA USA; 3 Chosun University, Dong-gu, Gwangju, Republic of Korea; 4 Greenbelt Endoscopy Center, Lanham, MD USA; 5 StatistX, Beer Tuvya, Israel

**Keywords:** Colonoscopy, Gastrointestinal cancer

## Abstract

Artificial intelligence (AI) has increasingly been employed in multiple fields, and there has been significant interest in its use within gastrointestinal endoscopy. Computer-aided detection (CAD) can potentially improve polyp detection rates and decrease miss rates in colonoscopy. However, few clinical studies have evaluated real-time CAD during colonoscopy. In this study, we analyze the efficacy of a novel real-time CAD system during colonoscopy. This was a single-arm prospective study of patients undergoing colonoscopy with a real-time CAD system. This AI-based system had previously been trained using manually labeled colonoscopy videos to help detect neoplastic polyps (adenomas and serrated polyps). In this pilot study, 300 patients at two centers underwent elective colonoscopy with the CAD system. These results were compared to 300 historical controls consisting of consecutive colonoscopies performed by the participating endoscopists within 12 months prior to onset of the study without the aid of CAD. The primary outcome was the mean number of adenomas per colonoscopy. Use of real-time CAD trended towards increased adenoma detection (1.35 vs 1.07, p = 0.099) per colonoscopy though this did not achieve statistical significance. Compared to historical controls, use of CAD demonstrated a trend towards increased identification of serrated polyps (0.15 vs 0.07) and all neoplastic (adenomatous and serrated) polyps (1.50 vs 1.14) per procedure. There were significantly more non-neoplastic polyps detected with CAD (1.08 vs 0.57, p < 0.0001). There was no difference in ≥ 10 mm polyps identified between the two groups. A real-time CAD system can increase detection of adenomas and serrated polyps during colonoscopy in comparison to historical controls without CAD, though this was not statistically significant. As this pilot study is underpowered, given the findings we recommend pursuing a larger randomized controlled trial to further evaluate the benefits of CAD.

## Introduction

Colonoscopy with removal of adenomatous and serrated polyps has significantly decreased the incidence of and mortality from colorectal cancer^1^. A meta-analysis reported that although colonoscopy was the most effective test for detection of colon polyps and cancer, the pooled prevalence of interval cancers was 3.7%^2^. Interval cancers frequently result from missed lesions such as adenomatous and serrated polyps. In a meta-analysis of tandem colonoscopy studies, the lesion miss rate has been reported to be as high as 26%^3^.

Novel endoscopic devices and technologies have been introduced to improve adenoma detection^4–7^. Recently, computer-assisted detection (CAD) and diagnosis have been proposed as potentially useful artificial intelligence (AI) applications in colonoscopy. Early studies demonstrated high sensitivity and specificity of polyp detection in an experimental or retrospective setting^8–11^.

To improve polyp detection, CAD should ideally be performed in real time. However, only a few studies have reported real-time CAD during colonoscopy. Klare et al. demonstrated that an automated polyp detection system was able to detect 55 of 73 polyps (75%) in real time^12^, confirming the feasibility of real-time CAD in a clinical setting. Wang et al. conducted a prospective randomized controlled study to compare the difference in adenoma detection rate with or without real-time automated CAD^13^. They demonstrated a higher adenoma detection rate with CAD (29% vs 20%, p < 0.001). The increase was due to a significantly higher rate of diminutive adenoma detection in the CAD group. Significant limitations of the study include the single-center design and the low prevalence of adenomas in the study population in China, leading to adenoma detection rates that are lower than the thresholds needed to meet quality metrics for colonoscopy at Western centers such as those in the United States where the prevalence of adenomas is higher. In a meta-analysis by Hassan et al. evaluating five randomized controlled trials including Wang et al., pooled ADR was significantly greater with CAD than with control (36.6 vs 25.2%, RR 1.44 [95% CI 1.27–1.62], *p* < 0.01)^14^.

The purpose of our study was to analyze the efficacy of a real-time CAD and determine whether the system could increase the detection of adenomatous polyps in a Western population.

## Methods

### Study population and design

This was a prospective single-arm, open-label pilot study with a historical control group. Patient enrollment and colonoscopies were conducted at two centers: Veterans Affairs Palo Alto Health Care System in California (Center 1), and Greenbelt Endoscopy Center, a private endoscopy unit in Maryland (Center 2). Two physicians performed the colonoscopies at Center 1, and four physicians performed the colonoscopies at Center 2. Each physician performed 50 study colonoscopies with the real-time CAD system. We enrolled patients undergoing elective colonoscopy regardless of indication (screening, surveillance or diagnostic) who were aged 40 years or older and had no prior history of colon resection, hereditary colorectal cancer syndrome, or inflammatory bowel disease. We excluded patients in which colonoscopy could not be completed due to poor bowel preparation. All enrolled patients in the study group provided informed consent. The historical control group consisted of 50 consecutive patients per physician who underwent elective colonoscopy within 12 months prior to onset of the study without the assistance of CAD. Similar to the study group, patients from the historical group were aged 40 years or older with no history of colon resection, hereditary colorectal cancer syndrome or inflammatory bowel disease. As in the study group, we excluded patients whose colonoscopies could not be completed due to poor bowel preparation. The study protocol was performed in accordance with the Declaration of Helsinki and approved by the institutional review board of Stanford University and each participating study center.

### Computer-aided detection (CAD) system

We used the EndoVigilant platform (EndoVigilant Inc, Maryland, United States) which can process 30 frames per second. This platform was was developed using 83,000 colon images extracted from video recordings of 300 colonoscopy procedures performed by 6 endoscopists across 4 different endoscopy centers in the US between July 2018 and July 2019. The colon images included multiple polyp views of each polyp varying in size, morphology and difficulty of detection as well as other non-polyp images of interest from the procedures. This data set was labeled and reviewed by trained personnel. The system was validated on a dataset containing 21,454 colon (polyp and non-polyp) images extracted from video recordings of 30 colonoscopy procedures different from those used for training data, a public database of 612 polyp-containing images from Hospital Clinic of Barcelona, Barcelona, Spain. Validation of the system on these datasets showed per-image sensitivity of 0.90 and a per image specificity of 0.97 and Area under curve (AUC) of 0.94. The EndoVigilant CAD system processed HD video feed from the endoscopy system over an SDI cable at 30 frames per second with a latency of 33–50 ms. The CAD system was connected to the endoscopy system without any changes or upgrades required of those systems. The CAD system output was displayed on a second monitor.

### Procedures

Colonoscopies were performed by six experienced endoscopists (> 5000 procedures previously performed by each) with high-definition colonoscopes (CF-HQ190 or PCF-H190, Olympus, Tokyo, Japan) and high-definition monitors. In the study group, the CAD system output was displayed on a second high-definition monitor that was placed adjacent to the primary endoscopy monitor, allowing the physician to conveniently view either screen. Insertion of the colonoscope and polyp resection were typically performed while viewing the primary monitor to avoid any potential detrimental effects due to lag time differences between direct video output from the endoscopy system and the augmented video output from the CAD system. Detected polyps were removed at the discretion of the endoscopist using snare or forceps in accordance with standard clinical practice and were sent for pathologic examination. Pathology review was performed by the Pathology Department at the Veterans Affairs Palo Alto Health Care System, and typically involved a gastrointestinal pathologist.

### Outcome measures

The primary outcome was the mean number of adenomas detected per colonoscopy in the study group versus control group. Secondary outcomes included the mean number of all neoplastic polyps (defined as adenomas and serrated polyps) (1), serrated polyps alone (2), and non-neoplastic polyps (hyperplastic, mucosal excrescence, normal tissue) (3) detected per colonoscopy, adenoma detection rate (4) and serrated polyp detection rate (5) in screening colonoscopies. Additional exploratory endpoints included evaluation of characteristics of the detected neoplastic polyps—size, morphology (only applied to polyps 6 mm or larger), and location (defined as proximal if the polyp was located from cecum to mid-transverse colon and distal from mid-transverse colon to rectum). Further exploratory endpoints included adenoma detection rate and serrated polyp detection rate. Nominal *p*-values were reported for the exploratory variables, but no statistical power was assumed in this pilot study.

### Statistical analysis

Continuous variables were summarized using sample size, mean, standard deviation, median, minimum, maximum and 95% confidence interval by group. Categorical variables were summarized using frequencies and percentages by group. Differences between the groups in continuous variables were tested for significance using the independent sample t-test. Differences between the groups in categorical variables were tested for significance using the chi-square test. In addition, stratified analysis was done for each category of colonoscopy indication (diagnostic, screening or surveillance) to separately test for differences between the groups. Given the one primary and five secondary endpoints utilized in the study, Bonferroni correction was applied for the secondary endpoints. We utilized an alpha value of 0.01 (0.05/5). Findings with *p*-value below 0.01 were considered statistically significant. Data was analyzed with R software version 3.6 (R Development Core Team, Vienna, Austria) by an independent statistician (StatistX, Beer Tuvya, Israel). A post-hoc power calculation was performed.

## Results

### Patients and basal characteristics

The mean age of the patients was 62.0 and 63.5 years in the CAD and control groups, respectively (p = 0.054). The percentage of females was 45% in the CAD and 43% in the control group (p = 0.68). Distribution by indication for colonoscopy was 50% vs 45% for screening, 35% vs 38% for surveillance, and 15% vs 17% for diagnostic in the CAD and historical groups, respectively. There was no difference between the groups in the indication for colonoscopy (p = 0.41). The mean procedure time was longer in the CAD group, 21.4 ± 9.1 min vs 19.5 ± 7.2 min (p = 0.004). The mean time for colonoscope withdrawal from the cecum to the anal verge in procedures where no polyps were identified was not statistically different between the CAD and historical groups: 9.1 ± 3.9 min vs 8.5 ± 2.7 min (p = 0.20) (Table 1).

**Table 1:** Patient characteristics.

	Study group	Control group	Significance
Number of patients (Center 1, Center 2)	300 (100, 200)	300 (100, 200)	–
Female (%)	134 (45%)	128 (43%)	NS (p = 0.68)
Age (mean ± SD)	62.0 ± 9.4	63.5 ± 9.6	NS (p = 0.054)
Indication: screening/surveillance/diagnostic	151/105/44 (50%/35%/15%)	135/114/51 (45%/38%/17%)	NS (p = 0.41)
Boston Bowel Prep Scale (mean ± SD)	8.0 ± 1.3	8.0 ± 1.3	NS (p = 0.69)
Total procedure time (minutes)	21.4 ± 9.1	19.5 ± 7.2	p = 0.004
Withdrawal time in colonoscopies with no polyps (minutes)	9.1 ± 3.9	8.5 ± 2.7	NS (p = 0.20)

### Polyp detection

The primary outcome of mean number of adenomas per colonoscopy was higher in the CAD than in the control group (1.35 vs 1.07, p = 0.099), though this did not achieve statistical significance. There was a trend towards a higher mean number of serrated polyps per colonoscopy were detected in the study group (0.15 vs 0.070, p = 0.023) and an increase in the detection of all neoplastic polyps (adenomas and serrated polyps) 1.50 vs 1.14 (p = 0.038), but these did not achieve the Bonferroni corrected statistical significance (Table 2).

**Table 2:** Polyps found during colonoscopy (values given as mean ± SD number of polyps found per colonoscopy).

	Study group	Control group	Significance
Adenomas	1.35 ± 2.2	1.07 ± 1.8	p = 0.099
Adenomas and serrated polyps	1.50 ± 2.3	1.14 ± 1.9	p = 0.038
Serrated polyps	0.15 ± 0.52	0.070 ± 0.31	p = 0.023
Non-adenomatous non-serrated polyps	1.08 ± 1.72	0.57 ± 1.07	p < 0.0001
Adenomas and serrated polyps ≥ 10 mm	0.20 ± 0.73	0.19 ± 0.74	p = 0.91*
Adenomas and serrated polyps 6–9 mm	0.32 ± 0.92	0.23 ± 0.59	p = 0.14*
Adenomas and serrated polyps ≤ 5 mm	0.98 ± 1.53	0.71 ± 1.27	p = 0.021*
Adenomas and serrated polyps in proximal colon	0.98 ± 1.55	0.80 ± 1.41	p = 0.13*
Adenomas and serrated polyps in distal colon	0.52 ± 1.15	0.34 ± 0.81	p = 0.027*
Polypoid (Paris I) polyps ≥ 6 mm	0.44 ± 1.2	0.35 ± 0.8	p = 0.31*
Flat (Paris IIa/b/c) polyps ≥ 6 mm	0.09 ± 0.3	0.09 ± 0.8	p = 0.95*

*Nominal *p*-values were reported for the exploratory variables, but no statistical power was assumed in this pilot study.

There was no difference in the rate of detection of neoplastic polyps ≥ 10 mm. There were greater 6–9 mm neoplastic polyps (0.32 vs 0.23, p = 0.14) and 1–5 mm neoplastic polyps (0.98 vs 0.71, p = 0.021) in the CAD group. Compared to historical controls, there were increased neoplastic polyps detected in the distal colon in the CAD group (0.52 vs 0.34, p = 0.027). There were similar neoplastic polyps identified between the two groups in the proximal colon (0.98 vs 0.80, p = 0.13). Further, there was similar detection of polypoid (Paris type I; 0.44 vs 0.35, p = 0.31) lesions or flat (Paris type II; 0.09 vs 0.09, p = 0.95) lesions 6 mm or larger in size in the two groups. Polyp morphology was not specified for polyps < 6 mm. There was statistically significant increase in non-neoplastic polyps found in the CAD group: 1.08 vs 0.57 (p < 0.0001).

Of the procedures done for colorectal cancer screening (patients with no prior history of neoplastic polyps), there was similar adenoma detection rate (43.7% vs 37.8%, p = 0.37) and serrated polyp detection rate (6.6% vs 5.9%, p = 1.00) between the CAD and the historical group (Table 3). In addition, for surveillance procedures (patients with a prior history of neoplastic polyps), there was similar adenoma detection rate (66.7% vs 59.7%, p = 0.35) between the CAD and historical group. However, there was higher serrated polyp detection rate in the surveillance patients of the CAD compared to the control group (17.1% vs 4.4%, p = 0.0043).

**Table 3:** Adenoma and serrated polyp detection rates (values given as percentage of procedures in which at least one adenoma or serrated polyp is detected, with 95% confidence intervals).

	Study group	Control group	Significance
Adenoma detection rate in screening colonoscopies	43.7% (36–52%)	37.8% (30–46%)	p = 0.37*
Serrated polyp detection rate in screening colonoscopies	6.6% (3–11%)	5.9% (2–10%)	p = 1.00*
Adenoma detection rate in surveillance colonoscopies	66.7% (58–76%)	59.7% (51–69%)	p = 0.35*
Serrated polyp detection rate in surveillance colonoscopies	17.1% (10–24%)	4.4% (1–8%)	p = 0.0043*

*Nominal *p*-values were reported for the exploratory variables, but no statistical power was assumed in this pilot study.

**Figure 1:**
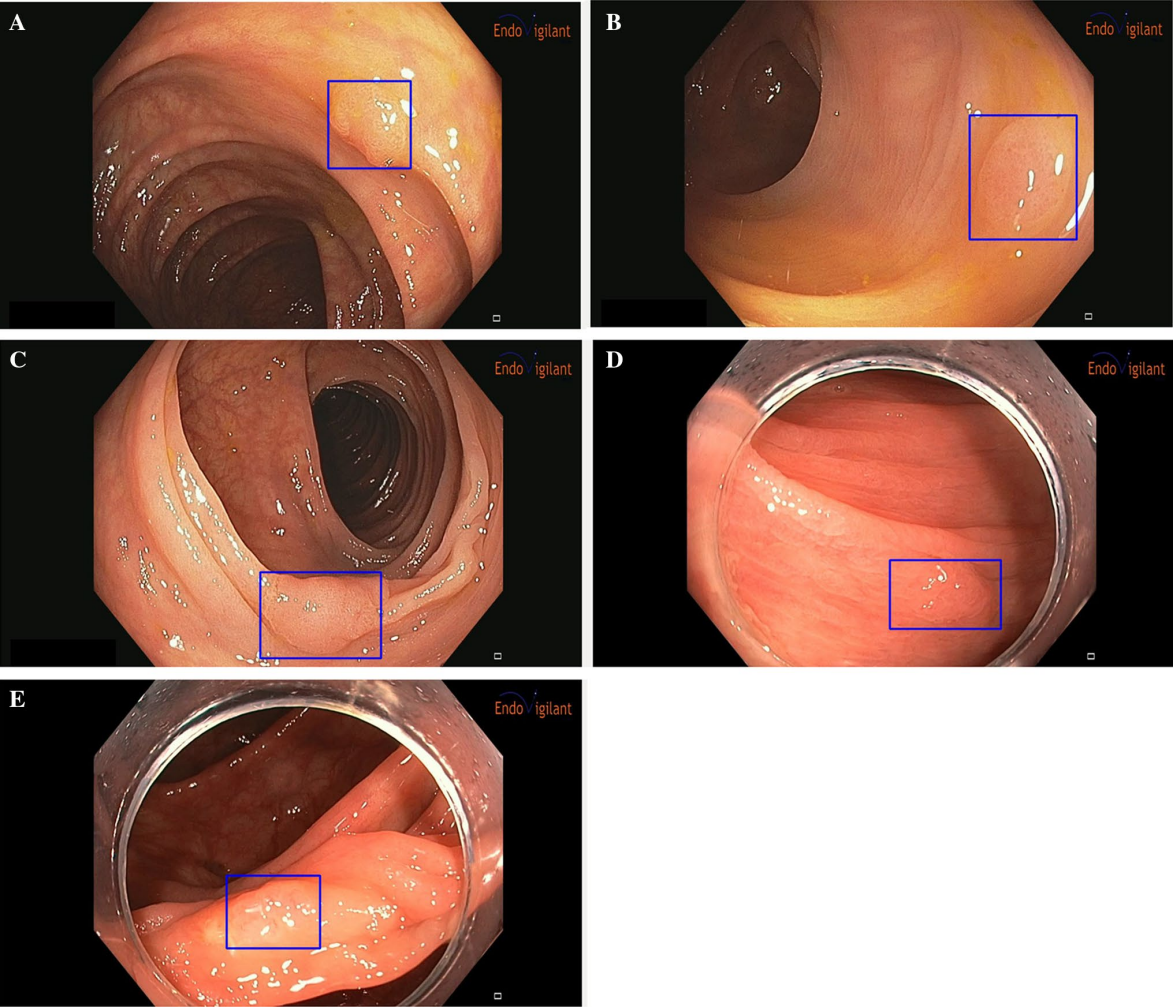
Representative examples of polyps detected using CAD system. (**A**) Proximal colon adenoma ≤ 6 mm. (**B**) Distal colon adenoma ≤ 6 mm. (**C**) Flat (Paris IIa) distal colon adenoma ≥ 10 mm. (**D**) Flat (Paris IIa) proximal colon serrated polyp 6–9 mm. (**E**) Flat (Paris IIa) proximal colon serrated polyp 6–9 mm with overexposure artifact due to surface reflection.

Representative images from the CAD output are shown in Fig. 1 and Video 1. Polyps that are detected by the CAD system are outlined with a rectangular box to call attention to the finding.

### Power analysis

Post-hoc power calculation based on primary outcome data showed the study was underpowered, with a power of 39.3%. To achieve a power of 80%, 663 patients would be required in each study arm.

## Discussion

AI applications in medicine have expanded rapidly in the past few years^15,16^. As colorectal cancer is the third leading cause of cancer-related death in the United States, an important clinical use case of AI is how it can help increase detection of pre-cancerous colonic lesions and decrease mortality. This application in colonoscopy can be most useful primarily on two fronts: polyp detection to improve adenoma detection rate by minimizing missed polyps, and polyp characterization to determine appropriate removal strategy including resect-and-discard, advanced endoscopic resection, or surgery^17^.

Unlike applying AI to analyze still images such as in pathology, radiology and dermatology, real-time support is required for AI to be clinically useful in colonoscopy. Polyps need to be detected during relatively fast-paced withdrawals in which the lesion is often not perfectly centered on the screen or under ideal lighting. The CAD system used in this study, from EndoVigilant, was trained on video recordings from hundreds of procedures at multiple sites across the United States. It utilizes a specialized class of deep learning algorithms called single shot detectors that are optimized for real-time performance and capable of highlighting multiple polyps in view in a single video frame. In a previous study, our group demonstrated that the system could detect 247/250 (98.8%) of polyps that were found by the endoscopist in 100 consecutive colonoscopies^13^.

In this study, we demonstrated that using real-time CAD during elective colonoscopy was associated with improved detection of adenomas, though this did not achieve statistical significance. As this was a pilot study, this result could be due to the low number of patients enrolled. A post-hoc power calculation showed the statistical power to be 39.3%. To achieve a power of 80%, 663 patients would be required in each study arm. Another possible explanation is the high historical mean adenoma detection rate of the six endoscopists involved in this study when compared to an adenoma detection rate benchmark of 25% for screening colonoscopies. From historical group data, the mean adenoma detection rate was 37.8% in screening colonoscopies and 59.7% in surveillance colonoscopies.

Interestingly, real-time CAD demonstrated a trend towards improved detection of neoplastic polyps (adenomas and serrated polyps). This increase was largely attributed to detection of polyps smaller than 10 mm. This was also due in part to a trend towards higher detection of serrated polyps, which are more subtle in morphology and can be easily missed. This suggests that a real-time CAD system may be useful in assisting gastroenterologists with small easy-to-miss lesions.

To our knowledge, this is the first prospective multicenter study to assess the clinical impact of real-time CAD in a North American population with a high prevalence of adenomas. Detection of adenomas and serrated polyps are important components of high-quality colonoscopy, and it has been reported that high adenoma detection rates may reduce the risk of interval cancer^14,15^.

By providing the endoscopist with a secondary monitor connected to the CAD system that displays the endoscopic view with any detected polyps outlined by boxes, it was possible to avoid any disruptions or alterations in the procedure. AI-enabled CAD systems typically have a short lag on the order of 100–125 ms due to re-capturing of video signals from the video feed and the AI software’s computational requirements. When performing complex maneuvers, such as polypectomy or insertion through a tortuous lumen, even a small lag can be distracting to the endoscopist. With the CAD output displayed on a secondary monitor, the endoscopist can use the system whenever convenient, which is typically while inspecting for polyps on scope withdrawal. Until further video connectivity and computing power efficiencies can be achieved to the point of offering a lag-free display, we believe endoscopists may prefer this configuration. As such, our study offers a realistic assessment of the potential benefits of using AI-based CAD in clinical practice.

The mean procedure time was slightly longer in the study group (21.4 ± 9.1 min vs 19.5 ± 7.2 min). As there were significantly more polyps detected using real-time CAD as compared to historical controls, it is reasonable to consider that a longer withdrawal time is mostly due to extra time spent removing polyps. In support of this, withdrawal times were found to be similar in both groups in patients who did not have any polyps. This also suggests that the difference in overall procedure time between groups was less likely due to endoscopists’ behavioral changes as a result of study participation. Given the importance of identifying and removing precancerous polyps, we believe the extra time spent is worthwhile. However, it is important to note that significantly more non-neoplastic polyps were found in the study group and this undoubtedly contributed to the longer procedure time. This highlights a need for additional training to help CAD systems better differentiate neoplastic from non-neoplastic lesions. By including six endoscopists at two separate centers, one academic and one private practice, our pilot study is more applicable to a broader audience than studies done only at major research institutions.

One limitation of the study is the use of a historical control group. However, the groups were well-matched and the historical group patients underwent colonoscopy within 12 months, a relatively short period in which there were no significant changes in equipment, referral or practice patterns. Another limitation is that the study was not blinded. We cannot exclude the potential effects of study participation on the endoscopists’ behaviors. The relatively small size of the study raises the possibility of type II statistical errors, particularly in measurements of outcomes where differences between the groups are likely to be relatively small. Indeed, several of our secondary outcomes such as adenoma detection rate had trends towards improvement with CAD that were not statistically significant. Finally, while study and historical colonoscopies were not matched by indication, there were similar proportion of colonoscopies classified as screening, surveillance, and diagnostic between the two groups.

As CAD systems evolve, future changes would include further improvements in accuracy and reduction in processing latency. It would be helpful to include additional features such as polyp characterization and classification, equipment detection, colon landmark detection and automated procedure reporting in the next generation of these systems^18,19^.

Based on our results, we recommend that a substantially larger randomized controlled study be performed to demonstrate a benefit with the use of CAD across multiple quality measures. Specifically, beyond assessing adenoma detection we recommend specifically exploring the impact of CAD on detection of serrated polyps. Additionally, a follow-up randomized controlled trial could more specifically evaluate the impact of CAD on endoscopists with historically low ADR.

## Conclusions

In the first prospective multicenter North American study, we demonstrated that using real-time CAD during elective colonoscopy was associated with improved though not statistically significant detection of adenomas. CAD was found to trend towards greater detection of serrated and neoplastic polyps. This pilot study has demonstrated need for larger randomized controlled studies to evaluate the impact of CAD on adenoma and serrated polyp detection as well as impact on endoscopists with lower ADR. We hope further studies can better elucidate the role of CAD within gastroenterology.

## Supplementary Information

Supplementary Legends.

Supplementary Video 1.
