# Supplementary material for: Clinical evaluation of a real-time artificial intelligence-based polyp detection system: a US multi-center pilot study
Source: Sci Rep. 2022 Apr 21;12:6598. doi: 10.1038/s41598-022-10597-y (PMC9023509; doi:10.1038/s41598-022-10597-y)
Supplement: Supplementary file 1 — Supplementary Legends. [file 41598_2022_10597_MOESM1_ESM.docx]

Video 1. Demonstration of colonoscopy using CAD.
